# The Selection of Remedies, and the Art of Prescribing Them

**Published:** 1863

**Authors:** Swayne Wickersham

**Affiliations:** Chicago, Ill.


ARTICLE IX.

### THE SELECTION OF REMEDIES, AND THE ART OF PRESCRIBING THEM.

BY SWAYNE WICKERSHAM, M.D., Chicago, Ill.

Read to the Chicago Medical Society.

I have selected this subject for my paper, in preference to any other of the multiplicity at my command, because, I know not how else I can occupy the little time that is allotted to me for preparation to such an advantage to the members of the Medical Society, as by the submission of my views regarding the improved methods for the administration of medicines, and the incalculable benefit that is to accrue to scientific medicine in consequence thereof.

It is not so much my desire to direct your attention to the selection of remedies therapeutically, as it is with a view to the eligibility of administration. Not but what the first consideration should take precedence of the second, but that the second claims equal thought and attention, in order to derive the greatest therapeutical benefit from any given medicament.

This is the age of progress: The coach and four is supplanted by the rail-car and steamboat, the news-boy by the telegraph, and the wooden ship by the iron-clad. The science of medicine is not at a standstill, but moves rapidly forward. It not only keeps pace with the collateral sciences, but takes the inside track, and goes on from time to time with accelerated speed. The public may doubt it, but no physician does, when he reflects upon the additional means at his command for diagnosing disease, and the corresponding simplicity and rationality of our therapeutics. Who among us but admits the inestimable value of auscultation and percussion; of the chemistry of the secretions and excretions; of the more nearly perfected symptomatology of morbid action; of the discovery of anesthetics; of the introduction of numerous medicines that are so much more certain in their curative influence, than those in use in former ages; of the methodical arrangement and treatment of diseases; and of the universal recognition of the *vis medicatrix naturæ?*

All these, by the best educated members of our profession, are recognized and acknowledged evidences of our advance. But what does the public say? They may tacitly admit the above, but will reproach us by the truthful assertion: that our remedies are equally uninviting, in fact, as repulsive, offensive, and unpalatable as those in use by the profession at a much earlier period. It is true we no longer prescribe distilled vipers, scorpions, frogs, lizards, worms, little dogs, mice and their teeth; tongues, livers, skulls, tails, claws, feathers, and brains of birds; shaving of men's skulls, after being put to violent death. But, do we prescribe remedies in a form any more pleasing to the vision, acceptable to the palate, and grateful to the stomach, than was done one hundred years ago? I think the interrogation will be answered in the negative, especially, when we call to our rememberance our copaiba and turpentine emulsions, our quinine, Dover, and alœtic powders, our dark hued and unsightly pills, our nauseous mixtures, and our bitter tinctures. We have gone higher and higher in all else appertaining to our avocation; but where is our improvement in this department? and echo answers, where? Notwithstanding, the general prevalence of an apathetic feeling among us, a few members of the profession have done much to cancel the defects, to bridge the chasm, to respond to the demand of the public, to win back the confidence of the people, to popularize legitimate medicine, and through this medium to assassinate the great popular quackery of the day,—homœopathy. I must say, though entertaining much respect for the profession, that the great body of medical men are, in this regard, dumb. They prescribe as if they were totally ignorant of the eligible pharmaceutical forms in which the medicine they prescribe can be and should be administered. The medical profession is one great family, and should be one great brotherhood; but, notwithstanding, the occasional absence of fraternal regard, we are so intimately associated, that we are, to an extent, common sufferers in the mishaps of each other,—what affects the interests of one is reflected, in a degree, upon the others. That which popularizes an individual as a physician, popularizes, to an extent, the great body, and *vice versa.* Hence, in the adoption of the improvements by each and all, we have a common interest. I cannot urge too strongly upon your attention, the demand of the public,—that we abandon our nauseous and offensive forms of administration, and give them the preparations of equal therapeutical value, in a form which is odorless, pleasant to the taste, and agreeable to the stomach.

Pharmaceutical eligibility is almost as essential in our prescriptions as it is to properly proportion the dose to correspond with the age, sex, temperament, and idiosyncrasies. I now proceed to bring to your attention some individual medicines in the pharmaceutical form, most desirable for administration, and will compare them with the form in which they are ordinarily ordered. I will commence with quinia sulphas. This is a medicine, I think I may say, in universal use among educated physicians. We cannot, at the present stage of medical science, abandon its use, and do justice to our patients. As an anti-periodic in our autumnal fevers, as a neutralizing agent in the malarial cachexias, as a sustaining medicine in typhoidal affections, we have not its equal, yet, how bitter to the taste. In solution, its extreme bitterness cannot be obviated. We direct a patient to take it and not to grumble. In powder, it cannot be incorporated with any vehicle that will conceal its objectionable feature. In pillular form, as made by the most accomplished members of the pharmaceutical profession of this city, it is disagreeable to many, refused by some, and unadapted to young children; and, yet I appeal to you if it is not the practice of ninety per cent of the physicians of this city to prescribe it as above-mentioned. For myself, I can say that I have almost abandoned the above forms, as prepared by our city pharmaceutists for the administration of this favorite medicine. I now almost universally direct it in the pillular form, as prepared by TILDEN & Co., New York. Their pills or granules are snowy-white, highly polished, small in size, and I firmly believe contain what they are represented to.

We cannot obtain a pill, manufactured in this city, that equals them. Our apothecaries are equally intelligent and accomplished as those of other cities, but they are destitute of the requisite machinery for their manufacture. I would draw no invidious comparison between the preparation described and the French one, which is similar to TILDEN'S, and is an elegant preparation. In this form, I have not the slightest trouble in getting patients to obey my instructions, when I desire to administer to them this medicine. It possesses all the elements to commend it to the eye, to the palate, and to the stomach. It is adapted, in the grain pill, to the infant, to the child, and to the adult. I now and then meet with a case where they tell me they cannot swallow a pill, I deny it, and tell them they can. I direct the medicine as stated, they take them and are pleased, and positively happy that they have but an odorless and tasteless medicine to swallow. Their acquired repugnance to unsightly pills yields to these. I have known children to desire the dose repeated, and to swallow them with avidity.

When I used the medicine as ordinarily ordered, I was much perplexed and annoyed by patients violating instructions and uttering manifold complaints, because the medicine was intensely bitter. A very desirable form, when given in powder as well as other unpalatable medicines, is to protect the taste by enclosing the substance in a wafer, but this method is not of general adaptation.

Allow me now to proceed to opium and its preparations. What I have said about the general use of quina sulphas, applies to this substance. We cannot do without it. Pain must be assuaged, inflammation cured, and sleep promoted; and you will search in vain for its equal to meet these indications. Now, will you continue to administer it in the powder, in the solution, in the pill, as prepared in our stores, or will you adopt the eligible form, the snowy-white and polished pill and granule of TILDEN & Co., or some other manufacturer who presents the same in the same form? Will you continue to disgust your patient with the medicine, and offend his stomach? He may thank you for relief, but he dislikes your medicine, and would not take it for the pleasurable sensation it gives him. Would it not be preferable, when we must give this medicine, to administer it in these little odorless and tasteless pills and granules, which are so acceptable to the sick, and to which objection will seldom be made. A few years' experience teaches me to speak with emphasis in the affirmative. The same eligible pharmaceutical form I adopt for the administration of all the more prominent medicines in use by us. A vast variety of these preparations are manufactured by TILDEN & Co., and are for sale at some of the stores of this city. I would mention the ferruginous preparations.—The plumbi acetas and opium, ipecac and opium, of each one grain, making a two-grain pill; the comp. cathar. pill of the U. S. Phar.; and, in fact, the medicine alone and in combination in the doses most in use among physicians; and by ordering a certain size, the dose can, in almost all instances, accommodate itself to the views of the practitioner.

These medicines are brought to this city in small bottles, containing one and five hundred pills or granules, and are dispensed as directed by physicians. I do not understand why physicians do not more frequently direct these medicines in the forms to which I have alluded.

The desirability must be admitted by all. To illustrate how seldom they are ordered, I would state that at an apothecary store in the south division of this city there were received 340 prescriptions in the month of September, 1862, not one of which called for these forms of medicine. A store in the west division, that does a large prescription business, at which I made inquiry has had the same result. The proprietor of a leading store in the south division informs me that there are but two of us who prescribe them from him.

I desire, finally, to consider the oleum terebinthinæ. This medicine is often prescribed, and, ordinarily, in emulsion. As an anthelmintic, as an important aid in certain stages of enteric fever, and as a medicine in some cases of sub-acute and chronic dysentery, it is invaluable. There are but few preparations that are so extremely nauseous to the majority of people; they, in most instances, take it, because we order it, and it does them good. They all complain, and some refuse to take it, and many cannot, in consequence of its producing emesis. The emulsive form, where it is possible, should be abandoned, and substituted by the pearls, containing about five drops, and weighing about three grains. These are tasteless, odorless, easily swallowed, and acceptable to the gastric organ. The emulsion is a favorite prescription of our much esteemed friend Prof. DAVIS, in connection with the tinc. of opium. I have used it, as advised by him, in numerous cases with the most happy effect. But why not gratify our patients by substituting one of the pearls and one of TILDEN'S granules, of one-eighth, one-sixteenth, or one-thirty-second of a grain of morphia sulphas, for the ordinary adult dose of the emulsion? I am certain, if either of us lay upon a sick-bed, which pharmaceutical forms of these medicines we would have given to us. If we did but give due attention to these things, the moral effect would be invaluable to the interests of legitimate medicine. I need not multiply. The illustrations that I have given are sufficient to prove that we are too careless and indifferent in the selection of our remedial agents, with regard to their pleasantness.

This is the age of quackery. I doubt not there are more people in America, especially in this latitude, who employ empirics, than at any former period. Lamentable as it is, yet I firmly believe that the confidence of the people is not in the regular profession, but in quackery, The history of medicine furnishes us with the evidence, that irregular practitioners have had an existence in all ages of our art, but it gives us no instance where the ordeal was so severe as that through which the profession is now passing. What has given the confidence of so many credulous people to that popular and formidable system of quackery that now obstructs the great medical highway? Upon what basis does it rest? Scientifically, it is foundationless, and has the least merit of any system that has ever been promulgated. The opinion of every observing and reflecting medical mind is, that it has but one merit, and that is the freedom from repulsiveness of their so-called remedies. I believe that it is almost useless to talk to the people upon subjects appertaining to our profession. We cannot convince them that a quack is a quack; that there cannot be but one true system of medicine; that there cannot be two sciences of the same thing. So long as these people have stomachs, my belief is, that the most potent weapon is the one that will reach the stomach. That is the spot where quackery must meet its foe. My arguments shall be used hereafter, so as to produce their effect in that locality. Prof. WOOD of Philadelphia says: "that the physician should be acquainted not only with the properties of medicines, and the diseases to which they are respectively applicable, but also with the art of prescribing them, so that they may be adapted to the peculiarities of individual patients; and by the mode in which they are administered, may produce the greatest curative effect with the least possible inconvenience."

It is from the neglect of the last clause of the quotation, that we are daily driving from us some one of our patrons, that he may associate with, and become a missionary to that objectionable system, that is gall and wormwood to all honest and learned physicians. Ask any one who employs homœopathists, what induced him to employ them; he will respond thus: in order to obviate the necessity of taking your disagreeable medicines. If the forms that I have mentioned be adopted by the profession, this reproach will no longer apply to us. We *can* give, in almost all instances, our medicine in a form that will be welcomed by our patients, and deprive them of the reason they assign for abandoning us. With these pharmaceutical forms at our command, no physician protects the interests of the profession, as he should, by permitting his patients to leave him in consequence of unpalatability of his remedies.

Let us select remedies that can be administered in small though efficient doses, and conceal the odor and taste by the improved means at our disposal. So far as the active principles of medicines have been discovered, we should give preference to them. Organic chemistry is very progressive, and is continually developing to us, upon what the medicinal virtue of our vegetable remedies depend. It behooves us, in whose hands legitimate medicine is to-day trusted, to lay aside the crude and the rough, and, where it is possible, to direct the essence, the active principles, which were not isolated and individualized, and, therefore, not at the command of our medical ancestors.

It is time that our decoctions and infusions were considered obsolete. It is time that our ordinary tinctures should be replaced by the concentrated ones. It is time that the disagreeable powder should be canopied by the wafer, or other means adapted to conceal its taste. It is time that a sick man should be treated by us as a sick man; we must cease to administer to his disgust. The sick child should no longer associate with the memory of the doctors' nauseous and repulsive medicines. When we have applied the principle of agreeable exhibition of medicine, which is the only life-spark of homœopathy, it will die a natural death, and have no resurrection.

*Similia similibus curanthur* and its infinitesimalism has been exploded. No scientific, no honest medical man gives credence to the utterances of its enunciators. Russia, after about forty years' experience with it, and granting it many hospital favors, has prevailed upon the Emperor to issue a decree forbidding a homœopathist to practice in his dominions. The hospital at Leipsic, in which this system was founded, died with SAMMY HAHNEMAN. The last one at London, also the last at Vienna, have ceased to have an existence. There no longer remain but three or four hospitals under their control upon the entire continent of Europe, and these have but a feeble and sickly existence. Denmark no longer has but three practitioners of that faith. Although several of the European governments have acted with their accustomed liberality to develop scientific truths, by granting the disciples of that faith an opportunity to prove their pretensions, by admitting them into the military hospitals; there is not a single instance on record where they did not signally fail. Such is homœopathy in Europe, after reaching about its fiftieth year of age. How is it in America? Honor to the United States Government for protecting the regular profession, by refusing the admission of the disciples of that false system into the army and navy, notwithstanding, Massachusetts sent her petitions, numerously signed, urging their official recognition; and Senator GRIMES of Iowa, was there to prostitute his intellect and insult the educated profession, by strongly urging the Government to grant their demands. Dr. PETERS of New York City, and editor of the late *North American Journal of Homœopathy*, who has written several works on homœopathic practice, and the recognized leader of them in the United States, and who has done more to popularize this system in America than any one hundred of his brethern, after becoming affluent, renounces this system, and deals fatal blows at this flimsy species of charlatanry. I will insert a few paragraphs, as found in his letter of renunciation, addressed to the editor of the *American Medical Times*, in 1861:—

"As far as lay in my power, I have not been unmindful for a day, from the commencement of my career as a medical student and practitioner, of the numerous brilliant advances in regular medicine, which have been constantly progressing both in this country and abroad. Infinitesimal doses are so repugnant to every fraction of common sense that I possess, that I have always felt absolutely degraded when making what I conceived to be necessary trials with them. I have always felt that I was doing something foolish or wrong when giving them, that I was dealing with quantities so minute and so powerless that it would be trifling with the lives of my patients to depend upon them in serious cases, and with their time and comfort in milder attacks. I have known many extraordinary instances of recovery from diseases and sickness in which no medicine had been given; and numerous consultations to which I was called by homœopathic physicians, in which severe disease had gone on unchecked by these powerless agents, more and more convinced me that they were irrational and unsafe." Thus wrote the only scholar they have had in the United States!

Soon after the publication of Dr. PETER'S article, Drs. FOWLER, BROWNE, and McDONALD, well-known homœopathic physicians in New York City and Brooklyn, published their renunciation in similar terms.

I have thus shown that this false system is on the shady side of life. They no longer have faith in their infinitesimalism. They to-day give more medicine in any given number of cases than we do. They select the identical pharmaceutical forms to which I have adverted in this paper. They call it homœopathy, and the people know no better. The part of a hypocrite is easily played with these medicines; and they will continue to flourish with then in private practice, until their use becomes general with us. It is astonishing to what an extent the public think that the homœopathists are the progressives. The people know not how free we are from routine-practice. They are ignorant of the wide eclecticism that governs us. They little think that each physician exercises, to an unlimited extent, his free agency, that we are no sectarians, no bigots; but that each physician is free to exercise his own intellect as to the kingdom of nature from which he shall call his remedial agents. That he gives or withholds medicines, directs in a small or large dose, as best accords with his judgment. I hold the homœopathists directly responsible for thus poisoning the public mind.

My own deep conviction is, that the only effectual way of annihilating these deep dyed scoundrels in our "day and generation," who, skunk-like, are too fetid to touch, but who poison the medical atmosphere by their odoriferous exhalations, is, for the regular profession in all cases where it is possible, to direct, administer medicine in the forms suggested in this paper.

